# Influence of Membrane Equivalent Weight and Reinforcement on Ionic Species Crossover in All-Vanadium Redox Flow Batteries

**DOI:** 10.3390/membranes7020029

**Published:** 2017-06-06

**Authors:** Yasser Ashraf Gandomi, Doug S. Aaron, Matthew M. Mench

**Affiliations:** 1 Electrochemical Energy Storage and Conversion Laboratory, Department of Mechanical, Aerospace and Biomedical Engineering, University of Tennessee, Knoxville, TN 37996, USA; yashrafg@utk.edu (Y.A.G.); daaron@utk.edu (D.S.A.); 2 Energy and Transportation Science Division, Oak Ridge National Laboratory, Oak Ridge, TN 37831, USA

**Keywords:** vanadium redox flow battery, crossover, equivalent weight, reinforcement, swelling, conductivity, UV-Vis spectroscopy

## Abstract

One of the major sources of lost capacity in all-vanadium redox flow batteries (VRFBs) is the undesired transport (usually called crossover) of water and vanadium ions through the ion-exchange membrane. In this work, an experimental assessment of the impact of ion-exchange membrane properties on vanadium ion crossover and capacity decay of VRFBs has been performed. Two types of cationic membranes (non-reinforced and reinforced) with three equivalent weights of 800, 950 and 1100 g·mol^−1^ were investigated via a series of in situ performance and capacity decay tests along with ex situ vanadium crossover measurement and membrane characterization. For non-reinforced membranes, increasing the equivalent weight (EW) from 950 to 1100 g·mol^−1^ decreases the V(IV) permeability by ~30%, but increases the area-specific resistance (ASR) by ~16%. This increase in ASR and decrease in V(IV) permeability was accompanied by increased through-plane membrane swelling. Comparing the non-reinforced with reinforced membranes, membrane reinforcement increases ASR, but V(IV) permeability decreases. It was also shown that there exists a monotonic correlation between the discharge capacity decay over long-term cycling and V(IV) permeability values. Thus, V(IV) permeability is considered a representative diagnostic for assessing the overall performance of a particular ion-exchange membrane with respect to capacity fade in a VRFB.

## 1. Introduction

Incorporating non-hydroelectric renewable energy sources (e.g., solar and wind power) into the electric grid requires robust and large-scale energy storage systems. Redox flow batteries (RFBs) are promising candidates for efficient, large-scale energy storage. Among many chemistries developed for RFBs, all-vanadium redox flow batteries (VRFBs) are the focus of this study. VRFBs are unique among all major chemistries developed for RFBs since they utilize the same ion (vanadium) in multiple oxidation states for negative and positive electrolytes; as a result, they do not suffer from irreversible capacity decay due to vanadium ion transport through the ion-exchange membrane [1]. VRFB systems were initially proposed by Skyllas-Kazacos and co-workers, and significant improvements have been made in increasing the power density and the performance via different research groups [2,3,4,5,6]. Typical VRFB systems have an energy density of 25–30 Wh·kg^−1^ and a round-trip efficiency of 70%–80%, depending on operating current. Recently, a high performance VRFB cell with up to 88% energy efficiency at a current density of 400 mA·cm^−2^ has been demonstrated successfully [2,7,8,9,10].

Although significant progress has been made in improving the performance of VRFBs, there are many issues yet to be addressed. One of the major remaining issues is the relatively rapid capacity decay during cycling compared to expected component lifetime. The primary contributions to capacity loss are undesired vanadium and water transport through the ion-exchange membrane, degradation of cell components (predominantly electrodes and membrane) and gas-generating side reactions [11,12,13]. Appropriate material selection, pretreatment protocol, and operating voltage range can largely avoid material degradation and side reactions. However, capacity decay due to vanadium and water transport across the membrane is inevitable. For VRFB systems, the following reactions are reported to occur when vanadium ions cross through the membrane [14].

(1) $$V^{2+}+2{\mathrm{VO}}_{2}^{+}+2H^{+}\to 3{\mathrm{VO}}^{2+}+H_{2}O$$

(2) $$V^{2+}+{\mathrm{VO}}^{2+}+2H^{+}\to 2V^{3+}+H_{2}O$$

(3) $${\mathrm{VO}}_{2}^{+}+V^{3+}\to 2{\mathrm{VO}}^{2+}$$

(4) $${\mathrm{VO}}_{2}^{+}+2V^{2+}+4H^{+}\to 3V^{3+}+2H_{2}O$$

Reactions (1) and (2) correspond to the case where vanadium ions of the positive electrolyte (V(IV) and V(V)) cross through the ion-exchange membrane and react with V(II) in the negative electrolyte. A similar case occurs when the vanadium ions of the negative electrolyte (V(II) and V(III)) travel through the membrane and react with V(V) in the positive electrolyte according to Reactions (3) and (4).

Several parameters contribute to capacity decay due to solute (vanadium ion) and solvent (water) transport through the membrane. The properties of the cell components (ion-exchange membrane, electrodes and flow-fields) and operating conditions (flow rate, temperature, vanadium and acid concentration, state of charge (SoC) and cell overpotential) are significant contributors to net transport.

Concentration gradient (diffusion) and electric field (via migration and electro-osmosis) significantly contribute to vanadium and water transport across the ion-exchange membranes. Water transport can also be affected by gradients in osmotic and hydraulic pressure [15]. The most common experimental approach for assessing the concentration-gradient induced crossover is via permeability cells, which has been used by several research groups [16,17,18]. The effect of various pretreatments of the membrane on the vanadium crossover has been investigated using a permeability cell [19]. Experimental approaches targeting the effect of the electric field on vanadium and water crossover are rare and usually require sophisticated experimental setups [15,17]. Therefore, assessing the effect of the electric field on crossover is more frequently studied via numerical modeling approaches [13,20]. Darling et al. introduced a new framework for modeling the crossover based on the charge-discharge capacity mismatch and defined the crossover current density using dilute solution theory [21].

It is also important to note that the transport of vanadium species through the ion-exchange membrane is influenced by electrolyte composition; thus, it is SoC dependent [15,22,23]. Recently, we reported on the effect of the electric field on vanadium crossover and deduced interaction coefficients for quantifying vanadium crossover as a function of SoC for Nafion^®^ (DuPont^TM^, Wilmington, DE, USA) membranes. As a result, the transport parameters for vanadium ions and water, with and without the effect of electric field and as a function of SoC, are now known for Nafion^®^ [15]. It has been shown that the permeability values of vanadium ions with different oxidation states through the ion-exchange membrane are dissimilar, and accordingly, as a function of cycling, vanadium ion accumulation is asymmetric between the negative and positive sides. This asymmetry results in imbalanced vanadium concentration in the negative and positive electrolytes over long-term cycling, causing capacity fade [15]. To understand this behavior, several modeling studies have been developed to simulate vanadium ion and water crossover through Nafion^®^ membranes [13,14,20,24,25,26,27,28,29]. Ultimately, however, more direct experimental tests are needed to confirm and validate any modeling study for these complex solutions and different membranes.

The ideal membrane should have low ohmic loss, high chemical, mechanical and thermal stability, and superior ionic selectivity. Maintaining high ionic conductivity favors the utilization of thin membranes in order to reduce ohmic overpotential, but necessarily increases diffusive crossover of vanadium ions and water, resulting in increased capacity fade as a function of time and concentration gradient. As a result, selection of an appropriate membrane for VRFB applications is a tradeoff between high ionic conductivity and decreased vanadium ion and water crossover.

Controllable properties of ion-exchange membranes include polymer type (anionic versus cationic), equivalent weight, and membrane reinforcement; the effects of these properties on vanadium ion crossover and capacity decay are of great importance. In addition, the membranes to be used for VRFB applications must meet several transport requirements to ensure economic and technical viability [30]. The incorporation of hydrophobic ionic liquids as membrane dopants has shown promising results for reduced vanadium ion and water crossover [31,32].

Perfluorosulfonic acid-based Nafion^®^ is a commonly-used membrane for VRFB applications. Although Nafion^®^ membranes have high mechanical and chemical stability, they have relatively high material cost and poor ionic selectivity. Considering the overall cost of a VRFB system, the ion-exchange membrane contributes 25%–45% of the total cost depending on energy capacity [33,34]. Furthermore, the overall cost of an ion-exchange membrane is correlated with equivalent weight (EW); thus, lower EWs are preferable from a material cost perspective. However, lower EW decreases the mechanical stability of the membrane among other changes.

The main motivation of the present work is to better understand the effect of equivalent weight and reinforcement on overall cell performance, ionic species crossover, and capacity decay. In this work, we report the results based on VRFB cells that used GORE-SELECT^®^ (Newark, DE, USA) or pure cast film membranes over a range of equivalent weight. Throughout this paper, “GORE-SELECT^®^” is conventionally used to indicate reinforced membranes, except in figures and tables where “GORE-SELECT^®^” has been used for labeling these types of ion-exchange membranes.

The test matrix includes in situ capacity decay experiments for different cell configurations under constant current density subjected to long-term cycling, polarization curves (obtained to demonstrate the cell voltage as a function of current density), AC impedance tests, ex situ membrane characterization and vanadium crossover tests.

## 2. Experimental Section

### 2.1. Membrane Selection and Pretreatment

The first set of membranes included non-reinforced cation exchange membranes with equivalent weights of 800, 950 and 1100 g·mol^−1^. The second set of membranes was reinforced cation exchange membranes with the same three equivalent weights as the non-reinforced membranes (800, 950 and 1100 g·mol^−1^). It is important to note that when the ion-exchange membranes are exposed to a solution containing vanadium, sulfuric acid, and water, they physically expand; it is necessary to measure this expansion to calculate accurate diffusive crossover rate constants. The details of such measurements have been provided in Section 4.

### 2.2. Method of Approach

**Figure 1:**
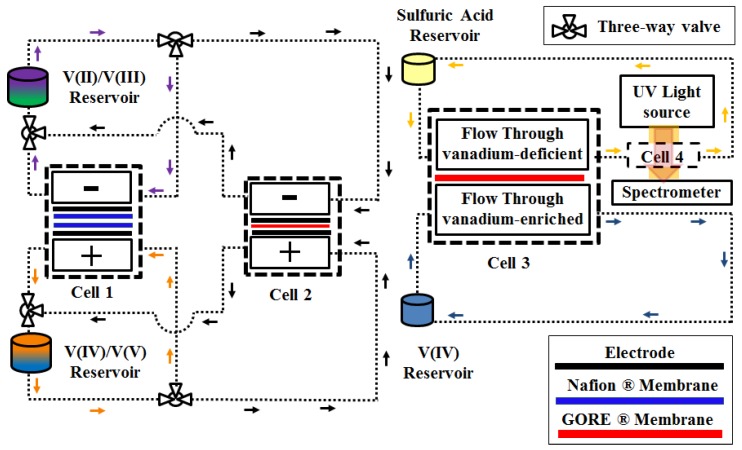
Schematic of the experimental setup.

A schematic of the experimental test system used in this work is in Figure 1. The test rig includes four single cells, two-channel peristaltic pumps (Cole Parmer, Masterflex L/S, Vernon Hills, IL, USA) and external reservoirs, as well as ultraviolet/visible (UV-Vis) spectrometers (THORLABS, Newton, NJ, USA) with light sources (Ocean-Optics, Dunedin, FL, USA). Furthermore, a central temperature control unit maintains a constant temperature of 30 °C for all of the cells and reservoirs.

The initial electrolyte of interest was prepared using Cell 1, which was a 25 cm^2^ active area cell. Pristine 39AA carbon paper electrodes (SGL Group, Wiesbaden, Germany) were sandwiched between a flow plate and the membranes. Two Nafion 117 membranes were utilized in Cell 1 in order to reduce the rate of crossover during electrolyte preparation. Two electrolyte reservoirs were utilized in conjunction with Cell 1 for V(II)/V(III) and V(IV)/V(V) couples. All reservoirs were sealed and under constant nitrogen purge while stir bars were utilized to ensure uniform composition of the reservoir solutions.

Cell 2 was used to record polarization curves and capacity decay data for the pure cast film and GORE-SELECT^®^ membranes. This cell utilized a 9 cm^2^ active area with single-path serpentine flow fields and 39AA (pristine, 1 layer) carbon papers as electrodes. Prior to conducting cycling experiments, pristine membranes (pure cast film or GORE-SELECT^®^ membranes) were immersed in a solution of 3.3 M sulfuric acid at room temperature for more than a week. A flow rate of 70 mL/min was supplied using a two-channel peristaltic pump. Cycling experiments were conducted at a current density of 100 mA/cm^2^ with cut-off voltage limits of 0.2 V and 1.9 V for discharge and charge modes, respectively.

In order to assess vanadium transport behavior, we have focused solely on the concentration-gradient induced crossover due to the number of ion-exchange membranes considered in this work. Utilizing concentration-gradient-induced crossover to represent actual crossover is common when many membranes are investigated [19] and/or the current density is low [15,21]. Readers are encouraged to refer elsewhere for a comprehensive analysis of electric-field-induced crossover [15,21].

Cell 3 was designed to investigate the concentration-gradient-induced V(IV) permeability for a series of different membranes. Cell 3 was a two-chamber 9 cm^2^ active area cell with flow-through flow fields and no electrodes. For Cell 3, the vanadium-enriched solution flowed from the V(IV) reservoir, and vanadium-deficient solution was circulated from the sulfuric acid reservoir and passed through a flow-through UV-Vis measurement unit cell (labeled as Cell 4 in Figure 1) to monitor the vanadium-deficient side’s solution in real time. The total time of electrolyte circulation was 24 h. During the circulation of vanadium-enriched and vanadium-deficient electrolytes, concentration gradient-driven crossover occurred from the vanadium-rich electrolyte to the aqueous sulfuric acid solution across the ion-exchange membrane of interest. The vanadium-deficient solution was directed to Cell 4 for real-time monitoring of V(IV) crossover using UV-Vis spectroscopy. To calculate the concentration of V(IV) on the vanadium-deficient side from the spectroscopic data, the spectra of standardized V(IV) with known concentration were recorded using the same parameters as the experiment. The real-time spectroscopic data were integrated and analyzed with scripts written in-house. This experimental procedure was repeated for different configurations of Cell 3 assembled using various pure cast film and GORE-SELECT^®^ membranes.

### 2.3. Testing Protocol

A multichannel potentiostat/galvanostat (Arbin Instruments, College Station, TX, USA) was employed to prepare electrolyte. The initial electrolyte was 1.5 mol/L VOSO_4_·*x*H_2_O (Alfa Aesar, Haverhill, MA USA) dissolved in 3.3 mol/L sulfuric acid (H_2_SO_4_). The initial volume of negative electrolyte was 50 mL, and the positive electrolyte was 100 mL; after initial charging at 1.8 V until reaching a cut-off current of 4 mA, 50 mL of positive electrolyte were removed to prepare equal volumes of negative and positive electrolyte at near-100% SoC. The electrolyte was discharged to 50% SoC at a constant 100 mA/cm^2^ to obtain the condition for polarization curves.

A single-channel Bio-Logic SP240 potentiostat (BioLogic Science Instruments, Seyssinet-Pariset, France) was used to conduct the charge-discharge processes and apply the desired overpotential or current. Cell 2, as shown in Figure 1, was utilized to conduct the polarization and cycling experiments. The cycling experiments were conducted at a constant current of 100 mA/cm^2^ for both discharging and charging until reaching cut-off voltages of 0.2 V and 1.9 V, respectively. AC impedance (at open circuit voltage) data were also obtained using the Bio-Logic SP240 potentiostat.

In order to assess membrane conductivity, Cell 2 was utilized at open-circuit and 50% SoC conditions using AC impedance. AC impedance experiments were conducted with the positive electrode serving as working electrode and a 5-mV perturbation amplitude; ohmic impedance was taken as the high frequency impedance with no imaginary components (high frequency *x*-axis intercept on a Nyquist plot). To estimate the contact resistance of the other cell components, a cell was assembled without any membrane, and the resistance was found to be ~0.03 Ω.cm^2^; and this magnitude was subtracted from the total high-frequency impedance to assess the ohmic impedance associated with the membrane.

### 2.4. UV-Vis Spectroscopy

To conduct the vanadium ion permeability tests, Cell 3 and Cell 4 were utilized. Vanadium-enriched solution was circulated in one side of Cell 3, while vanadium-deficient solution was circulated in the other side; the vanadium-deficient solution was directed to Cell 4 after leaving Cell 3 (refer to Figure 1). Therefore, two sets of electrolytes were prepared: one set for the vanadium-enriched side and one set for the vanadium-deficient side. For the vanadium-enriched side, the electrolyte was 1.5 mol/L VOSO_4_·*x*H_2_O (Alfa Aesar, Haverhill, MA, USA) dissolved in 3.3 mol/L sulfuric acid (H_2_SO_4_).

To conduct the V(IV) permeability measurements, it is necessary to minimize water transport across the membrane so that V(IV) permeability is only a function of its concentration gradient. To this end, the undesired driving forces of water transport (combined effects of osmotic and hydraulic pressure gradients) need to be balanced. Utilizing the symmetric configuration for Cell 3 (vanadium-deficient side versus vanadium-enriched side) prevents any hydraulic pressure gradient across the membrane. However, the osmotic pressure gradient must also be minimized. In order to balance osmotic pressure, multiple approaches have been described in the literature including matching sulfate concentration on both sides [15,17] or providing a counter cation on the vanadium-deficient side [35]. The use of a counter ion in the vanadium-deficient side introduces new complications to transport measurement and is not used in this work. Only sulfuric acid solution can be used in the vanadium-deficient side if the concentration of the sulfuric acid is chosen based on the procedure explained in our previous work [15]. Therefore, for the vanadium-deficient side, the sulfuric acid concentration was 4.8 M, resulting in a zero osmotic pressure gradient for the solvent across the membrane. The volumes of the vanadium-enriched and vanadium-deficient reservoirs were selected to be 200 and 80 mL, respectively.

The electrolytes used for VRFBs exhibit strong visible light absorbance. Therefore, UV-Vis spectroscopy can be utilized to assess the composition of the solution. In this work, the spectrophotometric measurements were made using spectrometers in a transmission configuration with a spectral range from 400 to 900 nm. Since the focus of this work is to assess the permeability of V(IV), electrolyte absorbance was measured at a 760-nm wavelength [15]. Such measurements allowed the application of the Beer–Lambert law to determine the relative concentration of V(IV) [15]. To obtain the spectra associated with the vanadium-deficient solution, real-time spectrophotometry was performed while the solution of the vanadium-deficient side was circulating through Cell 4, as shown in Figure S1 (see Supplemental Figure S1).

## 3. Vanadium Ion Permeability Assessment

To obtain the transport parameters for the different types of membranes, permeability values for V(IV) must be determined under a concentration gradient; based on that value, diffusive transport parameters for the particular membrane are formulated according to the procedure explained elsewhere [15]. Concentration gradient-driven crossover occurs from the vanadium-enriched side to the vanadium-deficient side across the membrane. The concentration of diffused vanadium ions is used to obtain the permeability values for a particular membrane [15,17]. The concentration of diffused V(IV) ions in the vanadium-deficient side can be described using the following equation.

(5) $$V_{v\_d}\frac{dC_{v\_d}\left(t\right)}{dt}=P_{V\left(IV\right)}^{m}\frac{A}{t_{m}}\left[C_{v\_e}-C_{v\_d}\left(t\right)\right]$$

In Equation (5), $V_{v\_d}$ represents the volume of the vanadium-deficient electrolyte reservoir; $C_{v\_d}$ is the concentration of the vanadium ion in vanadium-deficient electrolyte; $C_{v\_e}$ is the concentration of the vanadium ion of vanadium-enriched solution; A is the active area of the membrane in contact with solution; $t_{m}$ represents the thickness of the membrane (after equilibration with electrolyte); and $P_{V\left(IV\right)}^{m}$ is the permeability of the V(IV) through the membrane of interest. In Equation (5), the concentration of vanadium in the vanadium-enriched solution is assumed to be constant over the course of each vanadium permeability experiment (24 h), since the vanadium-enriched reservoir is relatively large compared to the vanadium-deficient reservoir. To calculate permeability values, Equation (6) can be integrated.

(6) $$\int_{0}^{C_{v\_d}}\frac{d(C_{v\_e}-C_{v\_d}\left(t\right))}{\left(C_{v\_e}-C_{v\_d}\left(t\right)\right)}=-\int_{0}^{t}\frac{P_{V\left(IV\right)}^{m}A}{V_{v\_d}t_{m}}\;dt$$

(7) $$\ln\left(\frac{C_{v\_e}}{C_{v\_e}-C_{v\_d}\left(t\right)}\right)=\frac{P_{V\left(IV\right)}^{m}A}{V_{v\_d}t_{m}}\;t$$

According to Equation (7), the trend of the left side of the equation versus time can be used to obtain the permeability. After obtaining the permeability values, diffusive transport parameters can be calculated based on Equation (8).

(8) $$\Lambda_{V\left(IV\right)}^{m}=\frac{P_{V\left(IV\right)}^{m}}{t_{m}}$$

In Equation (8), $\Lambda_{V\left(IV\right)}^{m}$ represents the V(IV) diffusive transport parameter.

## 4. Results

### 4.1. Capacity Decay during Long-Term Cycling

**Figure 2:**
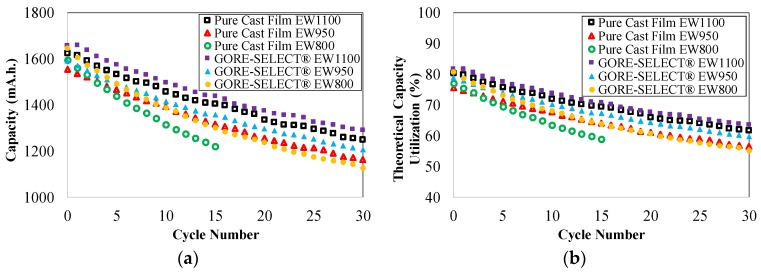
(**a**) Capacity decay as a function of cycle number; (**b**) theoretical capacity utilization as a function of cycle number. EW, equivalent weight.

Figure 2a includes the discharge capacity as a function of cycle number for the first 30 cycles for different pure cast film and GORE-SELECT^®^ membranes. In Figure 2a, the capacity decay data for the pure cast membrane with EW800 has only been reported for the first 15 cycles since the cell capacity became unstable due to membrane degradation. Figure 2b shows the theoretical capacity utilization for different membranes over the same duration. It is important to note that the theoretical capacity is determined using Cell 1 (from Figure 1) while charging the initial electrolyte; half of the total charge transferred to the electrolyte during the initial charge is evaluated as the theoretical capacity. Since both electrolytes were similar (1.5 mol/L V(IV) as VOSO_4_·*x*H_2_O (Alfa Aesar) dissolved in 3.3 mol/L H_2_SO_4_), half of the total initial charge corresponds to the full capacity window for an operating VRFB.

As shown in Figure 2a,b, the capacity and theoretical capacity utilization both decrease during cycling. The theoretical capacity utilization is an important metric for assessing overall vanadium ion utilization within a VRFB cell. Due to imposing voltage limits while maintaining cycling current (in this work, 0.2 V and 1.9 V), the theoretical capacity utilization is always lower than 100% since the total content of all dissolved vanadium ions cannot be utilized. The properties of the ion-exchange membrane directly affect the theoretical capacity utilization through the crossover rate. A higher rate of crossover decreases the open-circuit voltage (OCV) of the cell and consequently results in reaching the voltage limits sooner during a cycle, all other contributing parameters being equal. The theoretical discussion of such an effect is the focus of a previous publication [27]. According to Figure 2b, at the end of 30 cycles, the reinforced/EW1100 exhibits the highest magnitude of theoretical capacity utilization of ~64%, and the non-reinforced/EW800 shows the lowest theoretical capacity utilization (~59%, at the end of just 15 cycles). Furthermore, as seen in Figure 2b, increased equivalent weight results in increased theoretical capacity utilization for both reinforced and non-reinforced membranes. Comparing similar equivalent weights of non-reinforced and reinforced membranes, reinforcement increases the theoretical capacity utilization. For example, the EW1100 membrane showed 2% greater capacity utilization resulting from reinforcement; for the EW950 membrane, reinforcement increases the theoretical capacity utilization by 3% at the end of 30 cycles compared to non-reinforced.

**Figure 3:**
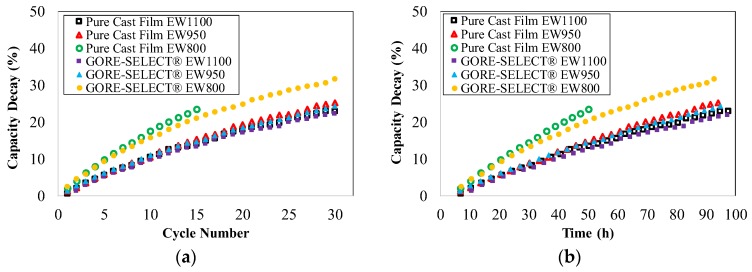
Capacity decay for different ion-exchange membranes as a function of (**a**) cycle number and (**b**) time.

Figure 3 includes the discharge capacity decay for different membranes as a function of cycle number and as a function of time. Here, the capacity decay is defined as the ratio of the decrease in discharge capacity to the initial discharge capacity of the battery.

According to Figure 3a, the reinforced EW1100 membrane exhibits the lowest rate of capacity decay (~22% at the end of 30 cycles), and non-reinforced EW800 shows the highest rate of capacity decay (~24% at the end of just 15 cycles). It is important to note that, although the number of cycles for all of the experimental membranes was 30 (except non-reinforced EW800), the total cycling time for the different configurations varied due to different ohmic overpotentials imposed as a function of membrane conductivity. However, in general, cycling experiments were conducted over the course of approximately 100 h. Similar to Figure 3a, the reinforced membrane with EW1100 exhibits the lowest rate of capacity decay (~22% at the end of ~100 h), and non-reinforced EW800 results in the highest rate of capacity decay (~24% at the end of ~59 h) as a function of time.

According to Figure 3, increased equivalent weight mitigates capacity decay for both non-reinforced and reinforced membranes. As shown in Figure 3, increasing the equivalent weight from 950 to 1100 g·mol^−1^ decreases the capacity decay by ~3% at the end of 30 cycles. The effect on capacity decay is more pronounced when increasing equivalent weight from 800 to 950 g·mol^−1^ for reinforced membranes in which the capacity decay decreased by 7% at the end of 30 cycles. Furthermore, it can be observed from Figure 3 that reinforcement decreases the capacity decay for identical EW membranes. Comparing the non-reinforced and reinforced membranes, for the case of EW1100 and EW950, reinforcement decreased the capacity decay by ~1.5% at the end of 30 cycles.

**Figure 4:**
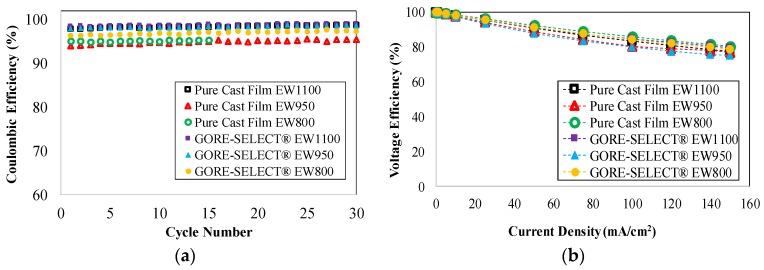

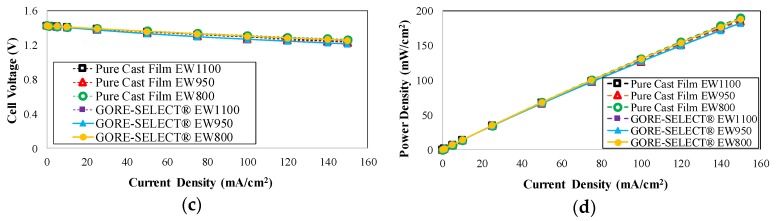
Characteristic plots for the experimental membranes: (**a**) coulombic efficiency, (**b**) voltage efficiency, (**c**) polarization curves and (**d**) power density.

The observed differences in capacity decay are primarily due to vanadium crossover since the other components (electrodes, flow fields and electrolyte) were kept consistent. Figure 4a includes coulombic efficiency for the membranes in this work. The coulombic efficiency was calculated here as the ratio of discharge capacity over charge capacity at 100 mA/cm^2^. The coulombic efficiency for all membranes tested in this work exceeded 95%. Figure 4b shows the voltage efficiency for the membranes over a range of current density. Voltage efficiency is defined here as the ratio of average discharging voltage over the average charging voltage up to 150 mA/cm^2^. As shown in Figure 4b, increasing the current density decreases the voltage efficiency for all experiments; voltage efficiencies exceeded 75% at 150 mA/cm^2^. Figure 4c shows polarization curve results at 50% SoC for cells based on each membrane. Figure 4d includes power density data for membranes as a function of current density for discharging conditions. As shown, a power density of 181–190 mW/cm^2^ was achieved at a discharge current density of 150 mA/cm^2^. Furthermore, it is important to note for clarity that the polarization curves and power density curves are shown just for discharge conditions since the charge and discharge cases were nearly symmetric.

It is important to note that, according to Figure 4a, coulombic efficiency for different membranes does not exhibit a direct correlation to increased equivalent weight or the addition of membrane reinforcement. The primary reason for such a trend lies in the definition of coulombic efficiency for VRFB systems. Coulombic efficiency is defined as the ratio of the discharge capacity over charge capacity [36]; therefore, for a membrane, both of these quantities can be small compared to the theoretical capacity while the cell still achieves high coulombic efficiency. Therefore, comparing Figure 4a with Figure 3, capacity fade as a function of cycle number or/and capacity decay as a function of time is a better metric to assess the viability of a particular membrane. Such a metric better represents superior ionic selectivity and the reduction of the vanadium crossover rate.

According to Figure 4, voltage efficiency and polarization curves do not show a linear correlation to either increased equivalent weight and/or membrane reinforcement. An ideal ion-exchange membrane would decrease vanadium ion crossover and ohmic overpotential simultaneously. However, if the reduction of vanadium ion crossover via use of a particular membrane is obtained at the cost of increased ohmic overpotential, the polarization curves and voltage efficiency (consequently the power density) show a negative impact, as evidenced by Figure 4b–d. This observation has been further clarified through measuring area-specific resistance (ASR) associated with each membrane.

Table 1 includes pre- and post-cycling ASR values for each pair of membranes at 100 mA·cm^2^ (see Supplemental Figure S2). For all experimental membranes, the ASR value increased during cycling (~9%–18%). For non-reinforced and reinforced membranes, increasing the equivalent weight yielded increased ASR: increasing equivalent weight from 800 to 950 g·mol^−1^ increased the ASR by ~36%, and increasing the equivalent weight from 950 to 1100 g·mol^−1^ increased the ASR by another ~16%. Considering the reinforced membranes, increasing the equivalent weight from 800 to 950 g·mol^−1^ increased the ASR by ~43%, while increasing the equivalent weight from 950 to 1100 g·mol^−1^ increased the ASR by a similar ~16%.

Furthermore, across all equivalent weights, reinforcement increased the ASR. Comparing the non-reinforced with reinforced membranes, for the EW1100 and EW950 sets, the ASR increases by ~18% due to membrane reinforcement, and for the EW800 pair, the increase in ASR is ~13%. In summary, the non-reinforced EW800 exhibits the lowest (0.15 Ω·cm^2^) and the reinforced EW1100 results in the highest value (0.28 Ω·cm^2^) of ASR.

A comparison of results in Table 1 and Figure 3 reveals that both of these membrane modification techniques (reinforcement and increased equivalent weight) resulted in decreased capacity decay; therefore, this simultaneous mixed effect is the primary reason for the trend observed for polarization curves, coulombic and voltage efficiencies and the power density graphs shown in Figure 4. This mixed effect has been further clarified in the next section. The increase in ASR during cycling results in an increase in ohmic overpotential as tabulated in Table 1 for the case of constant discharge at 100 mA·cm^−2^.

### 4.2. Crossover of Vanadium Ions

The major source for discharge capacity decay during cycling (as shown in Figure 3) is the undesired transport of vanadium ions through the ion-exchange membrane. In general, concentration gradient and electric field are the main driving forces for vanadium ion crossover. In this work, we have investigated the crossover of vanadium ions as a function of concentration gradient only. Among vanadium ions, concentration gradient-driven crossover has been assessed via focusing on the transport of V(IV) since the electrolyte preparation does not require any additional charge/discharge processes, ensuring uniform electrolyte composition.

**Figure 5:**
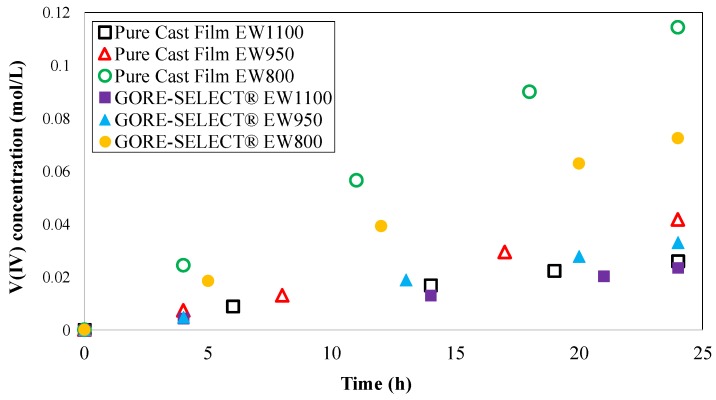
Concentration of diffused vanadium V(IV) ion to the vanadium-deficient chamber within the twenty-four hour time period.

Figure 5 shows the concentration of vanadium V(IV) ion on the vanadium-deficient side over a twenty-four-hour time period; the crossover behavior observed here corresponds to conditions at SoC = 0% on the positive side. The UV-Vis spectra were recorded at 6-h intervals (five sampling spectra) for each membrane, and the recorded spectra were utilized to calculate the concentrations shown in Figure 5.

As shown in Figure 5, the concentrations of diffused V(IV) to the vanadium-deficient side differ as a function of membrane composition. The maximum concentration of diffused V(IV) at the end of a 24-h crossover test was measured for the non-reinforced membrane with EW800 (~0.114 mol·L^−1^), and the minimum concentration was achieved by reinforced membrane with EW1100 (~0.023 mol·L^−1^). The concentration of diffused vanadium V(IV) ions shown in Figure 5 can be used to obtain the permeability values for different membrane morphologies using the slope of a semi-natural log plot, formulated in Equation (7).

**Figure 6:**
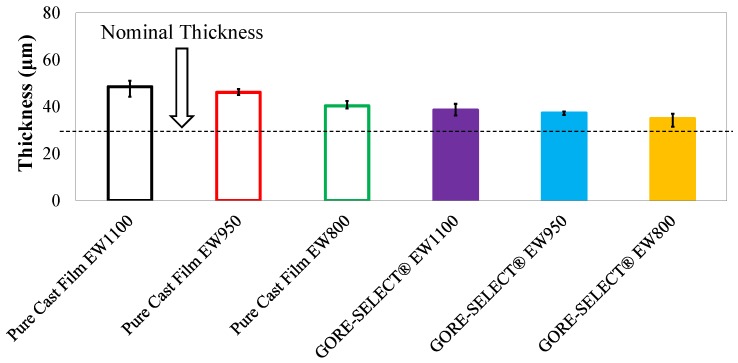
Thickness of the membranes after soaking in the solution of 1.5 mol/L V(III)/V(IV) and 3.3 mol/L sulfuric acid.

As indicated by Equation (7), the slope of semi-natural log plots (see Supplemental Figure S3) can be used to calculate permeability values; here, it is necessary to incorporate the expanded thickness of the membranes in the modeling framework. To this end, a solution of aqueous sulfuric acid and vanadium (3.3 mol/L acid, 1.5 mol/L vanadium (V(III)/V(IV) mix) was prepared, and the membranes were soaked in this solution for more than a week at room temperature. This composition was chosen to reflect the only stable vanadium species that can coexist in the membrane; V(II) and V(V) are expected to react with V(III) and V(IV). While the external solution is known to influence membrane properties, it is the internal environment that defines membrane properties. Figure 6 shows the thickness of the membranes measured after soaking in electrolyte. To measure the thickness, three sets of measurements were conducted using a digital micrometer (Mitutoyo, Kawasaki, Japan), and each reported thickness value was the average of all measurements; error bars were calculated based on the deviation of maximum and minimum thickness measurements from the average value.

As shown in Figure 6, the nominal thickness for all membranes tested was 30 μm. However, expansion (degree of swelling) differed significantly as a function of membrane properties. As a general trend, for both non-reinforced and reinforced membranes, the expansion was greater for higher equivalent weights. For example, among the non-reinforced membranes, EW1100 exhibits ~62% expansion in the through-plane direction, and EW800 shows ~35%. However, the reinforced EW1100 membrane expanded by ~29%, and reinforced EW800 expanded by ~16.5%. As expected, the addition of reinforcement significantly decreases through-plane expansion. The quantitative details of the cation exchange membranes tested in this work are shown in Table 2.

**Figure 7:**
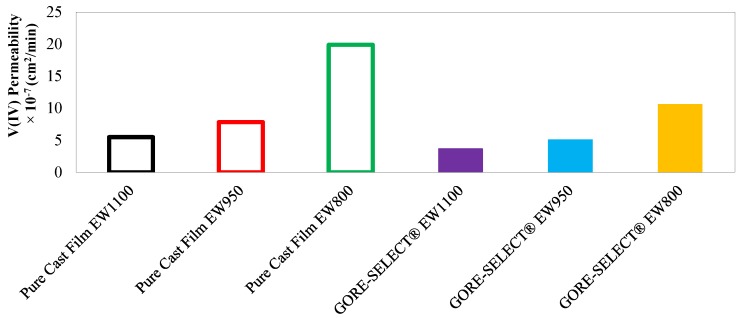
Measured permeability values for different membranes.

Figure 7 includes the resultant permeability values, based on the linear fits formulated in Equation (7). It is important to note that, for calculating the permeability values of V(IV) ions for each membrane, the thickness of the membranes equilibrated with the aqueous vanadium and sulfuric acid (as tabulated in Table 2) was utilized.

According to Figure 7, it is clear that, for both non-reinforced and reinforced membranes, increasing the equivalent weight decreases the V(IV) ion permeability. For non-reinforced membranes, decreasing the equivalent weight from 1100 down to 950 g·mol^−1^ increases the V(IV) permeability by 42%, and further decreasing the equivalent weight to 800 g·mol^−1^ results in 155% greater V(IV) permeability. In comparison, for reinforced membranes, decreasing the equivalent weight from 1100 down to 950 g·mol^−1^ increases the V(IV) permeability by 39%, and further decreasing the equivalent weight to 800 g·mol^−1^ further increases the V(IV) permeability by 109%.

Table 3 includes the numerical values of V(IV) permeability for each ion-exchange membrane, as well as the diffusive transport parameter, which has been formulated based on Equation (8). The diffusive transport parameter is utilized to calculate the concentration-gradient-induced crossover flux according to the mathematical formulation provided in our previous publication [15].

**Figure 8:**
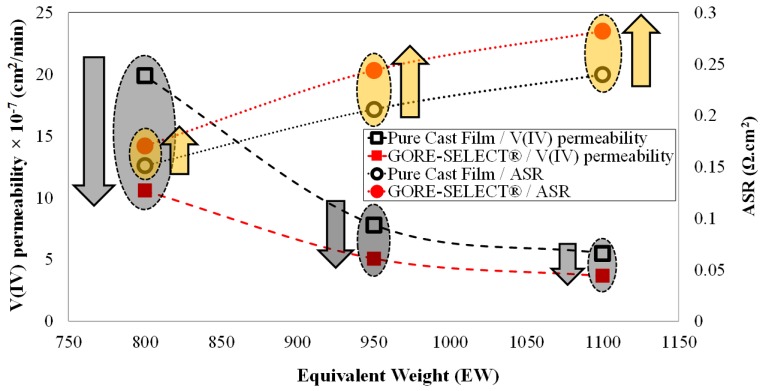
Correlation between equivalent weight with V(IV) permeability and ASR.

It is also important to quantify the effect of reinforcement on the reduction of V(IV) permeability. Figure 8 is a plot of V(IV) ion permeability and ASR as a function of equivalent weight. When comparing similar equivalent weights, reinforcement decreases the V(IV) permeability for all three compositions considered in this work. However, the effect is not linear. As shown in Figure 8, the observed trend for the ASR is the opposite of V(IV) permeability and exhibits an increase as a function of increased equivalent weight and reinforcement.

For the EW800 membranes, reinforcement decreased the V(IV) permeability by ~47%; for EW950, reinforcement decreased the V(IV) permeability by ~35%; and for EW1100, the reduction of V(IV) permeability as a function of membrane reinforcement was ~33%.

As discussed earlier, the primary reason for capacity fade as a function of cycling was assumed to be the crossover of vanadium ions. Therefore, to validate this assumption, it is critical to investigate any correlation between the discharge capacity decay and the V(IV) ion permeability values. As shown in Figure 3, the discharge capacity of cells with different configurations decreased as a result of cycling regardless of the type of membrane, but with different rates. No contribution is expected from side reactions since the voltage limits avoided their onset and also due to the lack of observed gas generation. In addition, component degradation should be negligible or consistent since all tests were conducted under identical conditions. Thus, the crossover of vanadium ions should be the primary driver of capacity fade.

**Figure 9:**
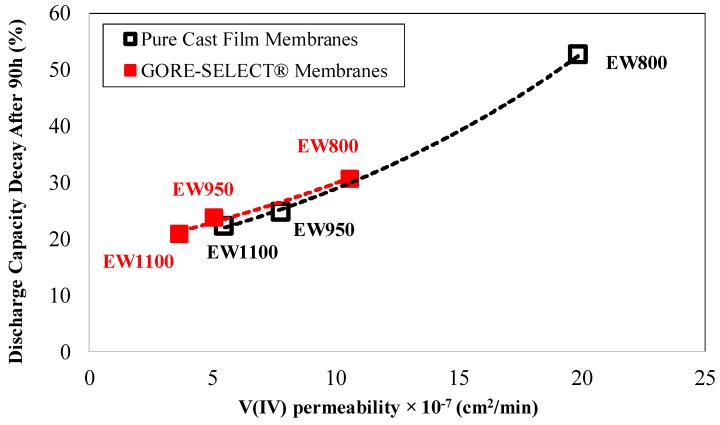
The discharge capacity decay at the end of cycling as function of vanadium V(IV) permeability.

Figure 9 includes the discharge capacity decay at the end of 30 cycles (or approximately 90 h) for non-reinforced and reinforced membranes as a function of vanadium V(IV) permeability. In Figure 9, the discharge capacity decay data associated with non-reinforced EW800 have been extrapolated since the cycling test for this membrane was only conducted for the first 15 cycles.

As is evident in Figure 9, there exists a strong correlation between the discharge capacity decay at the end of cycling with V(IV) permeability values. According to Figure 9, for non-reinforced membranes, increasing the equivalent weight from 800 to 950 g·mol^−1^, decreases the discharge capacity decay (after 90 h) from 53%–25% while decreasing the V(IV) permeability by 61%, and further increasing the equivalent weight to 1100 g·mol^−1^ decreases the discharge capacity decay to 22%, while the V(IV) permeability decreases by 30%.

A similar trend was observed for reinforced membranes: increasing the equivalent weight from 800 to 950 g·mol^−1^ decreases the discharge capacity decay from 31% down to 24%, while decreasing the V(IV) permeability by 52%; further increasing the equivalent weight from 950 to 1100 g·mol^−1^ decreases the discharge capacity decay to 21%, while decreasing the V(IV) permeability by 28%.

Finally, it is important to quantify vanadium crossover as a function of membrane thickness. As shown in Figure 6, the ion-exchange membranes selected for this work all had a nominal thickness of 30 μm; however, when soaked in a solution of aqueous sulfuric acid and vanadium, they swelled as a function of membrane reinforcement and equivalent weight. Given the permeability and swelling responses to EW, the following figure compares vanadium ion permeability (V(IV)) as a function of through-plane membrane swelling.

**Figure 10:**
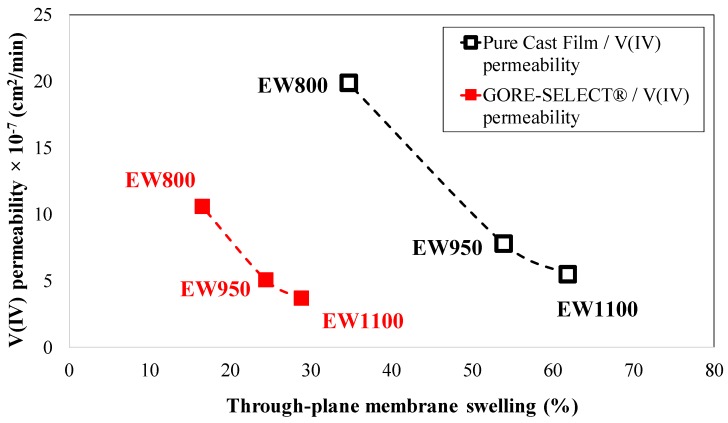
Correlation between V(IV) permeability and through-plane membrane swelling.

As shown in Figure 10, V(IV) permeability decreases for both the pure cast film and GORE-SELECT^®^ membranes as a function of increased through-plane membrane swelling. As tabulated in Table 2, increased equivalent weight increases through-plane swelling, and this results in reduced permeability for V(IV) ion. Therefore, an inverse correlation exists between swelling and permeability for pure cast film and GORE-SELECT^®^ membranes. The likely cause of this relationship is greater membrane thickness impeding V(IV) crossover.

A physicochemical description of the two distinct trends observed in Figure 8 is also of interest. First, as shown in Figure 8, increased equivalent weight results in decreased V(IV) permeability for both pure cast film and reinforced membranes. A macroscopic mathematical model based on conservation of mass, charge, momentum, and energy along with a meso-scale model based on dissipative particle dynamics (DPD) has previously been used to assess vanadium ion crossover behavior due to a concentration gradient [15,37]. Based on the macroscopic model of vanadium ion transport through polymeric membranes, vanadium crossover under the concentration gradient can be best described by the diffusive transport parameter [15]. As included in Table 3, the diffusive transport parameter decreases with increased equivalent weight. With a concentration-gradient-induced driving force for vanadium ion crossover, increased equivalent weight results in greater membrane swelling (as shown in Figure 6), resulting in higher diffusion resistance via increased path-length for the transport of vanadium ions; accordingly, the diffusive transport parameter is decreased. A meso-scale model description of vanadium ion transport has also been formulated by others based on DPD simulations, and it shows that increasing equivalent weight results in a stronger vanadium-sulfonate bond and accordingly more structured configuration. The stronger anion-cation interaction (V(IV) and sulfonate) as a function of increased equivalent weight, thus results in decreased diffusivity of vanadium ions [37].

As shown in Figure 8, membrane reinforcement results in decreased vanadium ion permeability for all equivalent weights. This observation is likely due to water transport behavior through the membranes; advection of vanadium ions through the membrane is influenced by water transport. It is well-established that water permeability through the membranes is both in equilibrium (solubility) and non-equilibrium (diffusivity) and is a strong function of the porous structure of the membrane [38,39,40,41]. Water transport has two distinct steps including surface adsorption (permeation through the surface) and internal absorption and transport across the membrane (diffusion); accordingly, the water transport rate is governed by water uptake and release rates [40]. The implementation of reinforcement decreases vanadium ion permeability by acting as a molecular sieve in the porous structure of the membrane [39]. The porous structure of the reinforced layer allows for the transport of water molecules while decreasing the transport of vanadium ions (V(IV)) by size exclusion. Accordingly, the contribution of vanadium ion transport via advection increases with decreased equivalent weight. Therefore, as shown in Figure 8, the inclusion of reinforcement results in a sharper decrease in vanadium permeability for lower equivalent weight membranes.

## 5. Summary and Conclusions

In this work, the effects of equivalent weight and membrane reinforcement on capacity fade and crossover in a VRFB system have been experimentally investigated. Three equivalent weights (800, 950 and 1100 g·mol^−1^) were considered for non-reinforced and reinforced membranes.

It is shown that vanadium crossover in VRFB systems can be mitigated either by increasing the ion-exchange membrane’s equivalent weight or implementing reinforcement. However, there is a tradeoff between decreasing capacity decay and increasing area-specific resistance (ASR), though this tradeoff is strongly dependent on equivalent weight. For non-reinforced membranes, increasing the equivalent weight (EW) from 950 to 1100 g·mol^−1^ decreases the V(IV) permeability by ~30%, but increases the area-specific resistance (ASR) by ~16%. Furthermore, increasing the equivalent weight from 800 to 950 g·mol^−1^ decreases the V(IV) permeability by ~61%, but increases the ASR by ~36%. Comparing the non-reinforced with reinforced membranes, for the EW1100 and EW950 membrane, the ASR increases by ~18% due to membrane reinforcement; but reinforcement decreases the V(IV) permeability by ~35% for EW950 membranes and by ~33% for EW1100 membranes. However, for the EW800 membranes, reinforcement decreases the V(IV) permeability by ~47%, but increases the ASR by ~13%.

Furthermore, it was shown that there exists a direct correlation between the discharge capacity decay over long-term cycling with V(IV) permeability values, confirming that the main contributor to capacity fade during cycling is due to vanadium crossover. As an example, for non-reinforced membranes, increasing the equivalent weight from 800 to 950 g·mol^−1^, decreases the discharge capacity decay at the end of cycling by ~53% and V(IV) permeability by ~61%. For reinforced membranes, increasing the equivalent weight from 800 to 950 g·mol^−1^ decreases the discharge capacity decay at the end of cycling by ~23% and V(IV) permeability by ~52%. This implies that V(IV) crossover tests can be utilized to assess the viability of a particular membrane of interest for long-term VRFB applications.

Finally, increased equivalent weight of the ion-exchange membrane decreases vanadium permeability, resulting in decreased capacity fade over cycling. When the membranes (pure cast film and GORE-SELECT^®^ membranes) are exposed to aqueous sulfuric acid and vanadium, they swell, but with different magnitudes. Increased equivalent weight increases the extent of swelling in both non-reinforced and reinforced membranes. As a result, the higher equivalent weight membranes impose extra resistance to vanadium crossover via the increased diffusion path-length, ultimately reducing capacity fade. Comparing the pure cast and GORE-SELECT^®^ membranes, reinforcement decreases the degree of membrane swelling. However, the degree of vanadium crossover mitigation significantly increases as a function of reinforcement, as well. Since the impact of reinforcement on permeability is greater than that of through-plane swelling, reinforced ion-exchange membranes with higher equivalent weight best mitigate vanadium crossover. The results of this study should provide deeper insight for ion-exchange membrane developers seeking optimized membrane structures for reduced vanadium ion and water crossover.

## Figures and Tables

**Table 1:** Initial and increase in area-specific resistance (ASR) and ohmic overpotential (at 100 mA·cm^−2^) as a function of cycling for different membranes.

Membrane Type	Initial ASR (Ω·cm^2^)	Relative Increase in ASR (%)	Initial Ohmic Overpotential at 100 mA·cm^−2^ (mV)	Increase in Ohmic Overpotential at 100 mA·cm^−2^ during 30 Cycles (mV)
Pure Cast Film EW1100	0.240	14.17	24.0	3.41
Pure Cast Film EW950	0.206	14.08	20.6	2.94
Pure Cast Film EW800	0.151	17.88	15.1	2.74
GORE-SELECT^®^ EW1100	0.282	16.67	28.2	4.66
GORE-SELECT^®^ EW950	0.244	12.70	24.4	3.11
GORE-SELECT^®^ EW800	0.171	16.96	17.1	2.93

**Table 2:** The details of the cation exchange membranes utilized in this work.

Membrane Type	Manufacturing Details	Nominal Thickness (μm)	Average Thickness after Soak (μm)	Through-Plane Swelling (%)
Pure Cast Film EW1100	Cast film	30	49	62
Pure Cast Film EW950	Cast film	30	46	54
Pure Cast Film EW800	Cast film	30	40	35
GORE-SELECT^®^ EW1100	Reinforced	30	39	29
GORE-SELECT^®^ EW950	Reinforced	30	37	24
GORE-SELECT^®^ EW800	Reinforced	30	35	16

**Table 3:** The vanadium V(IV) ion transport parameters through various ion-exchange.

Membrane Type	$P_{V\left(IV\right)}^{m}\;(cm^{2}\cdot min^{-1}\times 10^{-7})$	$\Lambda_{V\left(IV\right)}^{m}\;\left(cm\cdot min^{-1}\times 10^{-7}\right)$
Pure Cast Film EW1100	5.49	1131
Pure Cast Film EW950	7.80	1689
Pure Cast Film EW800	19.87	4919
GORE-SELECT^®^ EW1100	3.66	948
GORE-SELECT^®^ EW950	5.08	1361
GORE-SELECT^®^ EW800	10.59	3029

## Supplementary Materials

The following are available online at http://www.mdpi.com/2077-0375/7/2/29/s1: Figure S1: Calibration spectra for detection of the V(IV) ion via UV-Vis spectroscopy; Figure S2: Area-specific resistance (Ω·cm^2^); Figure S3: Semi-natural log plot used to determine permeability based on V(IV) ion diffused concentration.

## Nomenclature

$V_{v\_d}$	Volume of the vanadium-deficient electrolyte (m^3^)
$C_{v\_d}$	Concentration of V(IV) ion in vanadium-deficient electrolyte (mol/m^3^)
$C_{v\_e}$	Concentration of V(IV) ion in vanadium-enriched electrolyte (mol/m^3^)
$P_{V\left(IV\right)}^{m}$	Permeability of V(IV) ion through membrane *m* (m^2^/s)
$t_{m}$	Thickness of ion-exchange membrane (m)
A	Area of the ion-exchange membrane (m^2^)
$\Lambda_{V\left(IV\right)}^{m}$	Diffusive transport parameter of V(IV) ion through membrane *m* (m/s)
