# Supplementary material for: Influence of Membrane Equivalent Weight and Reinforcement on Ionic Species Crossover in All-Vanadium Redox Flow Batteries
Source: Membranes (Basel). 2017 Jun 6;7(2):29. doi: 10.3390/membranes7020029 (PMC5489863; doi:10.3390/membranes7020029)
Supplement: Supplementary file 1 [file membranes-07-00029-s001.pdf]

Supplementary Materials: Influence of Membrane Equivalent Weight and Reinforcement on Ionic Species Crossover in All-Vanadium Redox Flow Batteries

Yasser Ashraf Gandomi, Doug S. Aaron and Matthew M. Mench

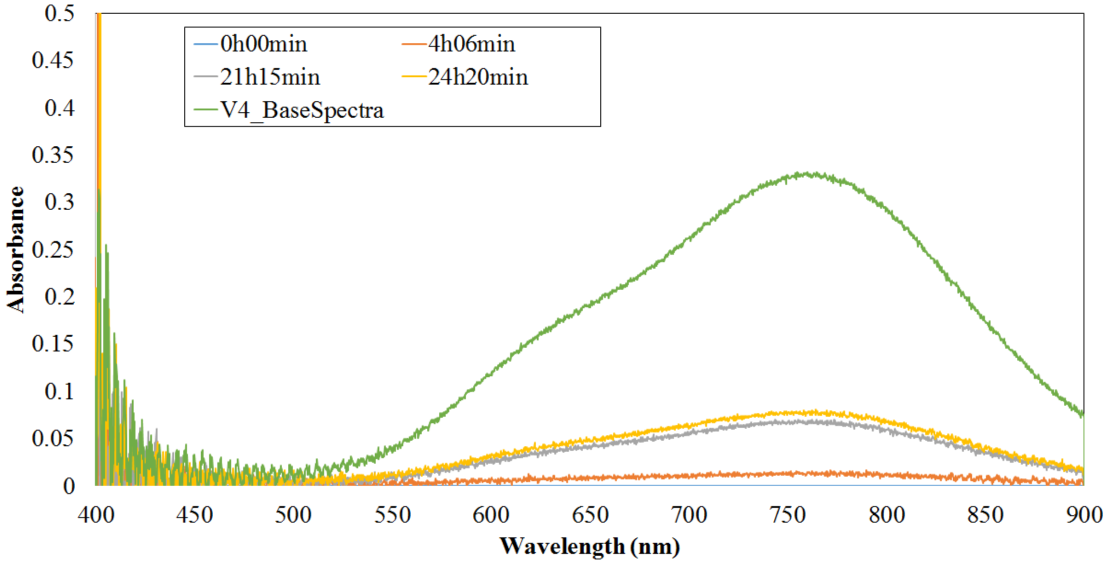

Figure S1. Calibration spectra for detection of the V(IV) ion via UV-Vis spectroscopy.

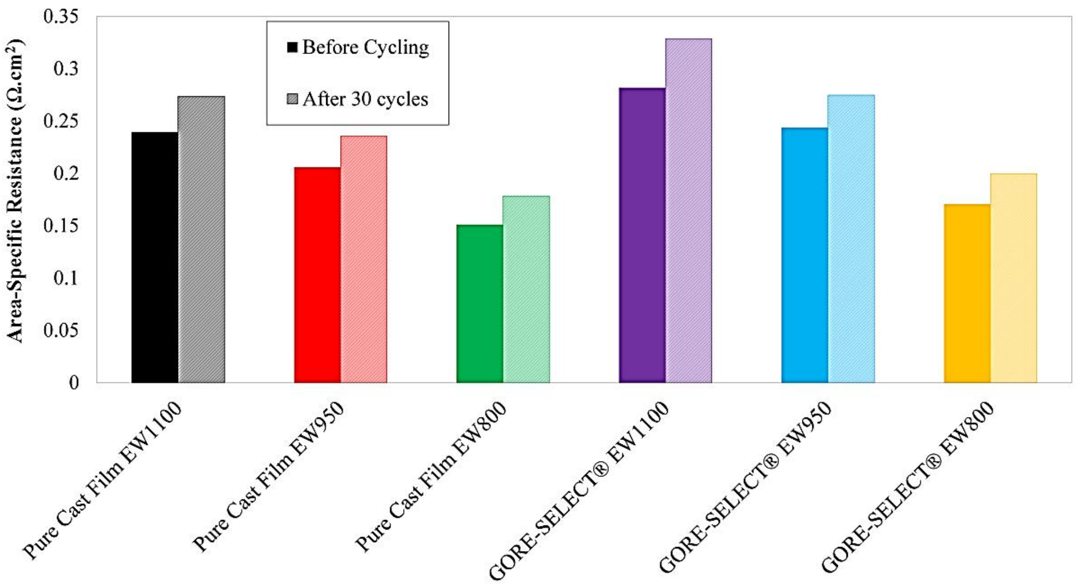

Figure S2. Area-specific resistance (Ω.cm²).

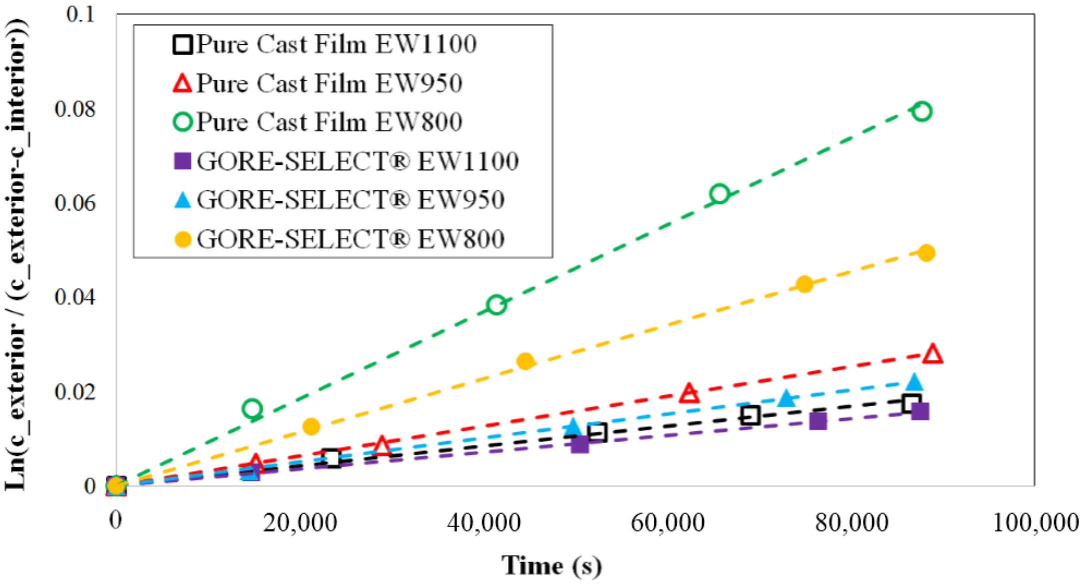

Figure S3. Semi-natural log plot used to determine permeability based on V(IV) ion diffused concentration.
